# Atypical Chemokine Receptor 3 Generates Guidance Cues for CXCL12-Mediated Endothelial Cell Migration

**DOI:** 10.3389/fimmu.2019.01092

**Published:** 2019-05-15

**Authors:** Chiara Tobia, Paola Chiodelli, Andrea Barbieri, Simone Buraschi, Elena Ferrari, Stefania Mitola, Giuseppe Borsani, Jessica Guerra, Marco Presta

**Affiliations:** ^1^Unit of Experimental Oncology and Immunology, Department of Molecular and Translational Medicine, School of Medicine, University of Brescia, Brescia, Italy; ^2^Unit of Biology and Genetics, Department of Molecular and Translational Medicine, School of Medicine, University of Brescia, Brescia, Italy

**Keywords:** *ackr3b*, angiogenesis, endothelial cell guidance, chemokine receptor, zebrafish

## Abstract

Chemokine receptor CXCR4, its ligand stromal cell-derived factor-1 (CXCL12) and the decoy receptor atypical chemokine receptor 3 (ACKR3, also named CXCR7), are involved in the guidance of migrating cells in different anatomical districts. Here, we investigated the role of the *ACKR3* zebrafish ortholog *ackr3b* in the vascularization process during embryonic development. Bioinformatics and functional analyses confirmed that *ackr3b* is a CXCL12-binding ortholog of human *ACKR3*. *ackr3b* is transcribed in the endoderm of zebrafish embryos during epiboly and is expressed in a wide range of tissues during somitogenesis, including central nervous system and somites. Between 18 somite and 26 h-post fertilization stages, the broad somitic expression of *ackr3b* becomes restricted to the basal part of the somites. After *ackr3b* knockdown, intersomitic vessels (ISVs) lose the correct direction of migration and are characterized by the presence of aberrant sprouts and ectopic filopodia protrusions, showing downregulation of the tip/stalk cell marker *hlx1*. In addition, *ackr3b* morphants show significant alterations of lateral dorsal aortae formation. In keeping with a role for *ackr3b* in endothelial cell guidance, CXCL12 gradient generated by *ACKR3* expression in CHO cell transfectants guides human endothelial cell migration in an *in vitro* cell co-culture chemotaxis assay. Our results demonstrate that *ackr3b* plays a non-redundant role in the guidance of sprouting endothelial cells during vascular development in zebrafish. Moreover, ACKR3 scavenging activity generates guidance cues for the directional migration of CXCR4-expressing human endothelial cells in response to CXCL12.

## Introduction

Chemokines and chemokine receptors, originally identified as mediators of the immune response, play non-redundant roles in various developmental processes (1). Among them, the chemokine receptor CXCR4 and its ligand stromal cell-derived factor-1 (SDF-1, also known as CXCL12) are involved in the guidance of cell migration in several anatomical districts, including neuronal patterning (2–4). Also, the CXCR4/CXCL12 system contributes to the development of vascular networks, including gut, kidney and retinal vascularization in mice (5–8), as well as lateral dorsal aortae (LDA) patterning (9) and hindbrain capillary network formation (10) in zebrafish (*Danio rerio*). In addition, chemokine signaling via *cxcr4a* and *cxcr4b* orchestrates the assembly of the lymphatic network in zebrafish embryo (11).

The atypical chemokine receptor 3 (ACKR3, also known as CXCR7) is a deorphanized receptor responsible for CXCL12 scavenging and internalization, with consequent generation of guidance cues for CXCR4-expressing cells in different organ systems (12). For instance, the two *ackr3* genes identified in zebrafish, named *ackr3a* and *ackr3b* (2), modulate cell guidance cues during posterior lateral line formation (13–16), trigeminal sensory neuron migration (17) and primordial germ cell migration (18) during embryonic development.

In vertebrates, two events characterize the development of the vascular system: vasculogenesis and angiogenesis. Vasculogenesis consists in the differentiation and migration of endothelial precursors to form a primordial vascular network, whereas angiogenesis is the development of new blood vessels from pre-existing ones. During vascular development in zebrafish embryos, angiogenesis in the trunk leads to the formation of the intersomitic vessels (ISVs) that occurs after the sprouting of endothelial cells from the dorsal aorta (DA) and posterior cardinal vein (PCV) originated by vasculogenic events (19). This complex process is finely regulated by several signaling pathways, including vascular endothelial growth Vegfaa (20), Notch/Dll4 (21), and Tie2 (22) signaling.

Here, we investigated the expression of the Cxcl12 scavenging receptor *ackr3b* in zebrafish embryo during development and the effect of its downregulation during the vascularization process of the embryonic trunk. Our results provide evidence that *ackr3b* plays a non-redundant role in the guidance of sprouting endothelial cells during ISV development in zebrafish. Accordingly, the CXCL12 gradient generated by ACKR3 expression in CHO cells guides human endothelial cell migration in an *in vitro* cell co-culture chemotaxis model.

## Materials and Methods

### Experimental Model and Subject Details

Wild-type AB and transgenic *Tg(kdrl:EGFP)* and *Tg(kdrl:EGFP;gata1:DsRed)* zebrafish lines were maintained at 28°C under standard conditions (23) and embryos were staged by hpf as described (24). To examine embryos older than 22 hpf, fish water was added with 0.2 mM 1-phenil-2-thiourea (PTU, Sigma-Aldrich, Saint Louis, MO, USA). For the observation of the *in vivo* phenotypes, embryos were anesthetized using 0.16 mg/ml Tricaine (Sigma-Aldrich).

### Bioinformatic Analysis

Zebrafish genomic sequences were analyzed using the University of California Santa Cruz (UCSC) Genome Browser (http://genome.ucsc.edu/) on the Zv9 (July 2010) *Danio rerio* assembly and the Ensembl zebrafish genome database (http://www.ensembl.org/Danio_rerio/Info/Index). Synteny analysis was achieved using the Synteny Database [PMID: 19465509]. Nucleotide and amino acid sequences were compared to the non-redundant sequences present at the NCBI (National Center for Biotechnology Information) using the Basic Local Alignment Search Tool (BLAST) [PMID: 2231712]. Multiple sequences alignment was performed using the MUSCLE algorithm [PMID: 15034147] using the following amino acid: human ACKR3, Acc. n° NP_064707; zebrafish, Acc. n° NP_001138286 (Ackr3a) and NP_001077301 (Ackr3b).

### Cell Lines

COS cells, grown in Dulbecco modified Eagle's medium (DMEM) (Gibco Life Technologies, Grand Island, NY, USA) supplemented with 10% FBS (Gibco Life Technologies), were transfected using Lipofectamin reagent (Invitrogen, Carlsbad, CA, USA) with a pcDNA vector harboring the *ackr3b* cDNA under the control of the CMV promoter and selected with geneticin (500 μg/ml; Invitrogen) to obtain *ackr3b*-COS cells. Stable expression of *ackr3b* was confirmed by RT-PCR. Human umbilical vein endothelial cells (HUVECs) were isolated from umbilical cords and used at early (I–IV) passages. HUVECs were grown on culture plates coated with porcine gelatin in M199 medium (Gibco Life Technologies), supplemented with 20% FBS, endothelial cell growth factor (10 μg/ml), and porcine heparin (100 μg/ml) (Sigma-Aldrich). Chinese hamster ovary (CHO) cells were transfected with a bicistronic pIRES-EGFP vector harboring the human *ACKR3* cDNA (kindly provided by Prof. Marcus Thelen, Institute for the Research Biomedicine, Bellinzona, Switzerland) using Lipofectamin reagent and selected with 350 μg/ml geneticin (Gibco Life Technologies) to obtain ACKR3-CHO cells. Stable expression of ACKR3 was confirmed by RT-PCR and fluorescent analysis of EGFP^+^ cells (see Figures 9A,B).

### CXCL12 Binding Assay

Mock and *ackr3b*-COS cells (2 × 10^5^ cells/experimental point) were incubated with 100 ng/ml of biotinylated human CXCL12 (_b_CXCL12) (R&D System, Minneapolis, MN, USA) for 1 h at 4°C or 37°C in 100 μl phosphate buffered saline (PBS), supplemented with 0.05% bovine serum albumin (Sigma-Aldrich), in the absence or in the presence of a 100x fold excess of unlabeled human CXCL12. Then, cells were fixed in 3% paraformaldehyde (PFA) for 10 min on ice and incubated with 50 ng/ml of avidin-FITC (Molecular Probes, Eugene, OR, USA). When indicated, cells were permeabilized with 1% saponin (Sigma-Aldrich) after fixation. Finally, samples were subjected to FACS analysis (MACSQuant, Miltenyi Biotec, Bergisch Gladbach, Germany).

### Chemotaxis

For chemotaxis experiments, linear gradient μ-Slide Chemotaxis chambers (Ibidi GmbH, Martinsried, Germany) were used following manufacturer's instructions. Briefly, HUVECs (2.5 × 10^6^/ml) suspended in M199 medium *plus* 3.5% FBS were seeded in the observation area at the center of the μ-slide chamber and allowed to adhere for the following 4 h. Then, the left reservoir of the observation area was filled with 50 ng/ml CXCL12 in M199 medium *plus* 3.5% FBS, whereas the right reservoir was filled with M199 medium *plus* 3.5% FBS alone. As negative controls, both reservoirs were filled with M199 medium *plus* 3.5% FBS alone or added with 50 ng/ml CXCL12. For co-culture experiments, the side reservoirs of the chamber were added with mock-CHO or ACKR3-CHO cells (3 × 10^5^/ml) with or without 50 ng/ml CXCL12. HUVECs were imaged every 15 min by time-lapse microscopy over a 13 h-period at 5-fold magnification under standard conditions with an Axiovert 200M fluorescence microscope (Zeiss, Oberkochen, Germany) and images were imported into ImageJ software (NIH, Bethesda, USA). 30–50 randomly chosen cells were tracked for each experiment using a manual tracking plug in (Fabrice Cordelières, Institut Curie, Orsay, France) and analyzed with chemotaxis and migration tool (Ibidi GmbH) to create trajectory plots of cell migration. In addition, the chemotactic parameters center of mass (COM, corresponding to the average of all cell positions at the end of the migration experiment) and x- and y- axes forward migration indexes (FMI, representing the efficiency of forward migration of cells parallel or perpendicular to the gradient, respectively) were calculated in each experiment.

### RT-PCR Analysis

Total RNA was isolated from CHO cells using TRIzol® Reagent (Invitrogen) according to manufacturer's instruction. Two micrograms of total RNA were retrotranscribed and 100 ng of cDNA were used for semi-quantitative RT-PCR analysis. The following primers were used:

*ACKR3*: forward 5′- cctgctctacacgctctcct-3′, reverse 5′- ggatattcacccagaccacca-3′;

*GAPDH*: forward 5′- catggccttccgtgttcctac-3′, reverse 5′- ttgctgttgaagtcgcaggag-3′;

### Whole-Mount *in situ* Hybridization (WISH)

Digoxigenin-labeled RNA probes were transcribed from linear cDNA constructs (Roche Applied Science, Penzberg, Germany). WISH was performed on embryos fixed in 4% PFA as described (25). For sectioning, zebrafish embryos were post-fixed in 4% PFA after WISH, dehydrated in ethanol series, cleared in xylol and paraffin embedded overnight.

### Morpholino Injection

Two independent morpholinos (Gene Tools, Philomath, OR, USA) targeting the ATG region of *ackr3b* were injected at the indicated concentrations in 1–4 cell stage embryos (*ackr3b*-MO1: 5′-ctcatcttggtccgtctttgttatc-3′; *ackr3b*-MO2: 5′-atcattcacgttcacactcatcttg-3′). *cxcr4a* morpholino: 5′agacgatgtgtccgtaataagccat-3′ (9). Standard MO (std-MO: 5′-cctcttacctcagttacaatttata-3′) was used as control.

### Phalloidin Staining

Phalloidin staining of zebrafish embryos was performed as described (26). Briefly, manually dechorionated embryos were fixed in 4% PFA for 3 h at room temperature (RT). Then, embryos were washed three times in PBS/0.1% Tween 20 (PBST) for 10 min and incubated in blocking solution (10% goat serum, 2% BSA, 0.5% Triton X-100 in PBS) for 2 h at RT. Embryos were incubated for 3 h at RT with Alexa Fluor 594 Phalloidin (1:400 in blocking solution, Thermo Fisher Scientific). Unbound antibody was removed by several PBST washes.

### Microscopy

Live and whole-mount hybridized embryos were photographed on agarose-coated dishes using either an epifluorescence Leica MZ16 F stereomicroscope (1X Plan Apo objective, NA0.141) (Leica, Wetzlar, Germany) equipped with digital camera or an Axio Zoom.V16 fluorescence stereomicroscope (Zeiss). Phalloidin-stained embryos were acquired on an Axiovert 200M fluorescence microscope (Zeiss) equipped with ApoTome.2 to enhance resolution. Evaluation of ISV defects was carried out on developing vessels in the region of the trunk above the prolongation of the yolk, as indicated in Figure 4A.

Confocal analysis of filopodia was performed using a LSM510 laser scanning microscope (Zeiss). To this purpose, 26–28 hpf embryos were fixed overnight with a PBS-based solution containing 1% PFA, 0.1% glutaraldehyde and 3% sucrose and mounted on glass slides with Mowiol 4.88 (Sigma). For consistency reasons and in order to minimize stage-related discrepancies, filopodia evaluation was carried out on the ISV pair in which the first vessel had already reached the roof of the trunk and the adjacent vessel was still growing up.

## Results

### Analysis of *ackr3* Genes in Zebrafish

The Gene and HomoloGene databases at NCBI [PMID: 25398906] indicate the presence of two putative co-orthologs of the human *ACKR3* gene in zebrafish, namely *ackr3a* (also named *cxcr7a*) and *ackr3b* on chromosomes 9 and 6, respectively. The analysis of the chromosomal regions surrounding the human and zebrafish genes, carried out using the Synteny Database [PMID: 19465509], showed a conserved synteny between the human chromosome 2 region harboring the *ACKR3* gene and *Danio rerio* chromosomes 6 and 9 regions where the two co-orthologs are located (Figure S1A). Similar to the human gene, both *ackr3a* and *ackr3b* zebrafish co-orthologs are organized in two exons, the second exon harboring the entire protein-encoding region (data not shown). In addition, multiple sequence alignment indicates a high level of identity of the human ACKR3 protein with zebrafish Ackr3a (51.1%) and Ackr3b (54.7%) polypeptides (Figure S1B).

Analysis of the Expressed Sequence Tag (EST) database indicates that the zebrafish genes are expressed at different levels, with 47 vs. 3 EST sequences corresponding to *ackr3b* and *ackr3a* transcripts, respectively. As shown in Figure S1C, the less abundant expression of *ackr3a* was confirmed by RNA-Seq data from the Wellcome Trust Sanger Institute Zebrafish Transcriptome Sequencing Project [PMID: 22798491, (27)]. In addition, the absence of RNA-Seq reads from 2-cell stage embryos indicates that the two genes are not maternally expressed whereas their expression can be detected from 3 to 6 h post fertilization (hpf) onward (data not shown).

On these bases, given the higher levels of expression and identity with the human counterpart, we focused our observations on the role of the *ackr3b* gene in vascular development.

### Cloning and Characterization of *ackr3b*

**Figure 1:**
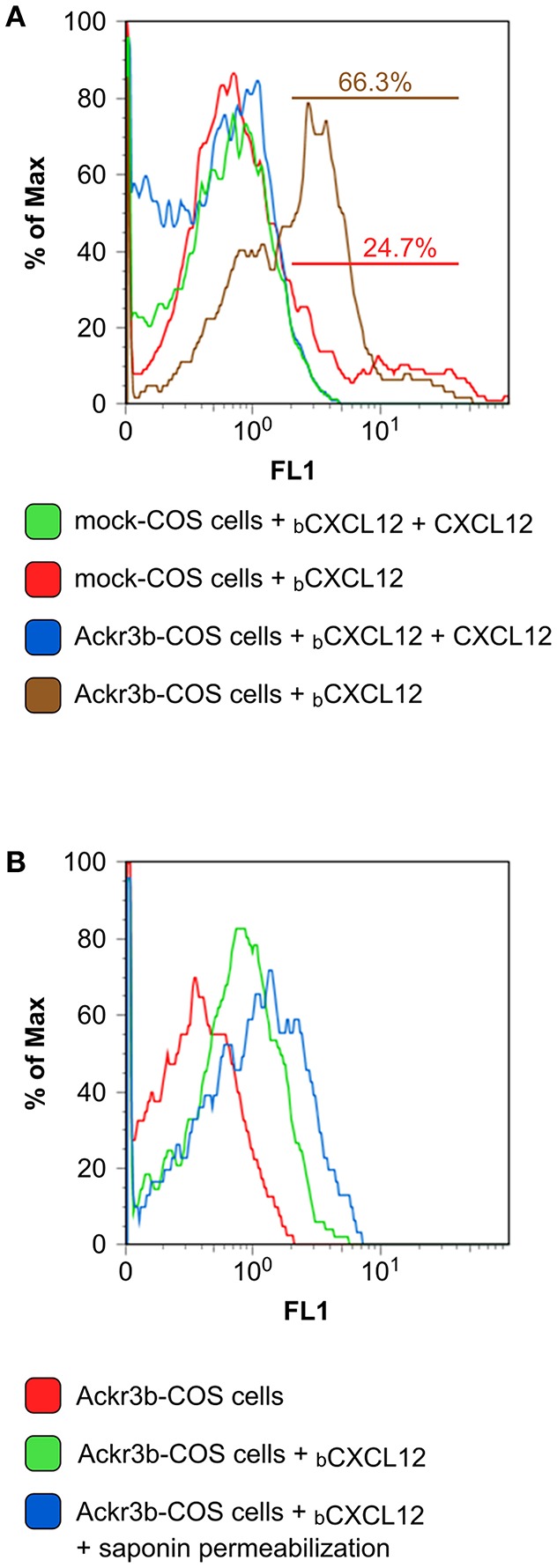
Zebrafish Ackr3b binds CXCL12. **(A)** FACS analysis of mock and Ackr3b-COS cells incubated for 1 h at 4°C in the absence or presence of biotinylated CXCL12 (_b_CXCL12). **(B)** To assess Ackr3b-mediated CXCL12 internalization, Ackr3b-COS cells were incubated for 1 h at 37°C in the absence or presence of _b_CXCL12 and permeabilized with saponin before FACS analysis. The results are representative of two independent experiments.

The complete coding sequence of *ackr3b* was amplified from total RNA isolated from zebrafish embryos at 55 hpf and cloned in the pcDNA3 expression vector (pcDNA3-*ackr3b*) to generate Ackr3b-overexpressing COS cell transfectants (Ackr3b-COS cells). To assess the ability of the Ackr3b receptor to bind its ligand CXCL12, Ackr3b-COS cells were sequentially incubated with biotinylated CXCL12 followed by avidin-FITC and subjected to FACS analysis. In a first set of experiments, mock and *ackr3b*-transfected cells were incubated with biotinylated CXCL12 in the absence or in the presence of an excess of non-biotinylated CXCL12. The incubation was performed at 4°C to prevent CXCL12 internalization. Under these experimental conditions, a significant increase in the percentage of cells able to specifically bind CXCL12 was observed in *ackr3b*-transfected vs. mock cells (66.3 and 24.7%, respectively), the limited binding of the chemokine to mock cells being possibly due to its interaction with cell surface glycosaminoglycans and/or with scarcely expressed endogenous ACKR3 or CXCR4 receptors (Figure 1A). In addition, incubation at 37°C of Ackr3b-COS cells with biotinylated CXCL12 followed by cell fixation, saponin permeabilization and FACS analysis indicates that the interaction of CXCL12 with Ackr3b induces ligand internalization (Figure 1B). Previous observations had shown the capacity of Ackr3b to bind and internalize zebrafish Cxcl12a (16, 18). Our results confirm that *ackr3b* is a zebrafish ortholog of the human *ACKR3* gene and demonstrate that the encoded receptor is able to bind and internalize also human CXCL12.

### *ackr3b* Expression During Zebrafish Embryo Development

**Figure 2:**
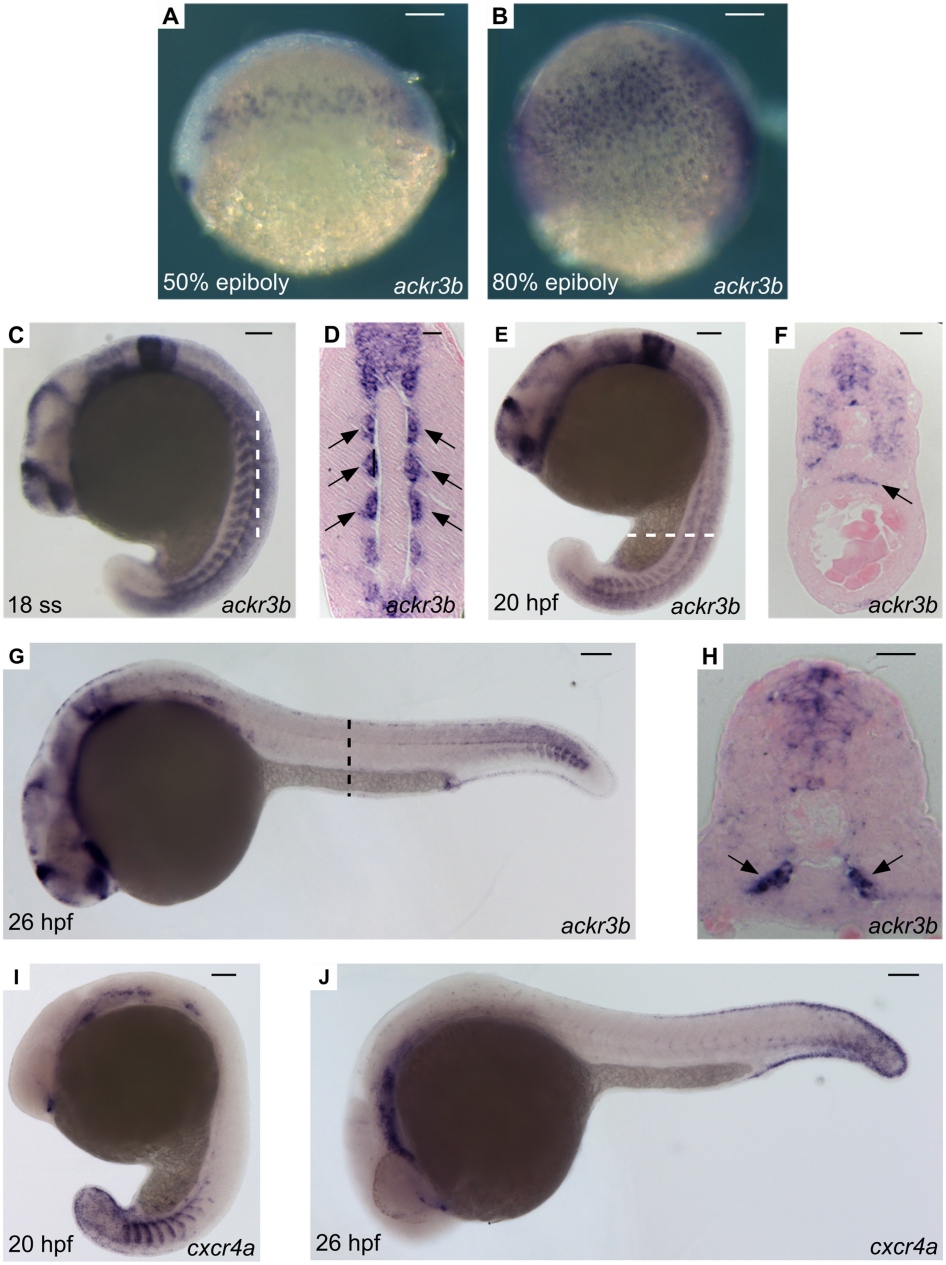
WISH analysis of *ackr3b* and *cxcr4a* expression during zebrafish embryo development. WISH analysis of *ackr3b* **(A–H)** and *cxcr4a* **(I–J)** mRNA expression was performed at the indicated stages of development. **(A–C,E,G,I,J)** Lateral view of whole-mount embryos. **(D)** Coronal cross section through the trunk highlighted as a dashed vertical bar in **(C)**, anterior to the top; arrows indicate somitic *ackr3b* expression. **(F,H)** Transverse cross sections through the trunk highlighted as dashed bars in **(E,G)**, respectively. Arrows in **(F**,**H)** indicate *ackr3b*-positive developing gut and the basal part of the somites, respectively. **(A–C,E,G,I,J)** Scale bar: 100 μm. **(D,F,H)** Scale bar: 25 μm.

A thorough investigation of *ackr3b* spatio-temporal expression pattern in zebrafish embryos was carried out employing both RT-PCR and whole-mount *in situ* hybridization (WISH) analyses followed by paraffin embedding and sectioning. In keeping with RNA-Seq data (see above), *ackr3b* is expressed in zebrafish embryo from 3 hpf onward, whereas no expression was observed in 2–4 cells embryos (data not shown). In agreement with previous observations (28), the *ackr3b* transcript is found in the endoderm of zebrafish embryos between 50 and 80% epiboly in a characteristic salt and pepper manner (Figures 2A,B). During somitogenesis (18 ss), the gene is expressed in a wide range of tissues, mainly in the central nervous system (rhombomeres 3, 5, and 6, hindbrain, midbrain, spinal cord and floor plate) and in somites (Figure 2C). As highlighted in coronal cross sections, somitic expression of *ackr3b* is restricted to the inner part of the somites, in close proximity to the notochord (arrows in Figure 2D). At 20 hpf, the *ackr3b* transcript is broadly detectable in the somites (Figure 2E) and a strong expression is evident at the level of the developing gut under the axial vasculature (arrow in Figure 2F). At 26 hpf, *ackr3b* expression is maintained in the central nervous system and somitic expression becomes particularly evident in the caudal region (Figure 2G). Moreover, transverse cross sections revealed that the strongest *ackr3b* expression is restricted to the basal aspect of the somite around the axial vessels (arrows in Figure 2H). By 48 hpf, the a*ckr3b* transcript is detectable in the branchial arches and in the lateral line organs (neuromasts), whereas expression in the neural tube is lost and becomes restricted to the floor and roof plates (data not shown).

### Downregulation of *ackr3b* Impairs ISV Guidance

Similar to *ackr3b, cxcr4a*, and its ligands *cxcl12a* and *cxcl12b* are expressed in the somites during zebrafish early somitogenesis (29). Then, *cxcr4a* is expressed in endothelial cells of ISVs and DA starting from 20 hpf (Figure 2I), to fade in a cranio-caudal fashion by 26 hpf (Figure 2J). This suggests that Ackr3b may provide vascular guidance cues in the Cxcr4a/Cxcl12 interplay during developmental angiogenesis in the trunk of zebrafish embryo. To assess this hypothesis, we used an antisense morpholino (MO) knockdown approach (30) and designed a first MO directed against the 5' UTR spanning the *ackr3b* ATG start codon (*ackr3b*-MO1). Dose-response experiments indicated that the mortality rate in embryos injected with an optimal dose of MO, equal to 0.2 pmoles/embryo, was 9-10 and 5–6% for *ackr3b*-MO1 and control std-MO, respectively. Surviving embryos were then grouped in different phenotypic classes based on their morphology (Figures S2A–E). In all the experiments, embryos with severe and very severe phenotypes were discarded (72/206 in three independent experiments) whereas embryos with mild (84/206) and close-to-normal phenotypes (50/206) were used for further analysis and considered together.

The *ackr3b* gene plays a non-redundant role in primordium migration in zebrafish embryos (13). On this basis, in order to validate the *ackr3b*-MO1 treatment herewith adopted, wild type AB zebrafish embryos were injected with 0.2 pmoles/embryo of *ackr3b*-MO1 and analyzed at 48 hpf by WISH using a specific *claudin b* probe. In keeping with previous observations (13), *ackr3b* morphants showed an impaired neuromast migration (6/7 *ackr3b*-MO1 vs. 0/9 std-MO), thus confirming the effective knockdown of the gene (Figures S2F,G).

**Figure 3:**
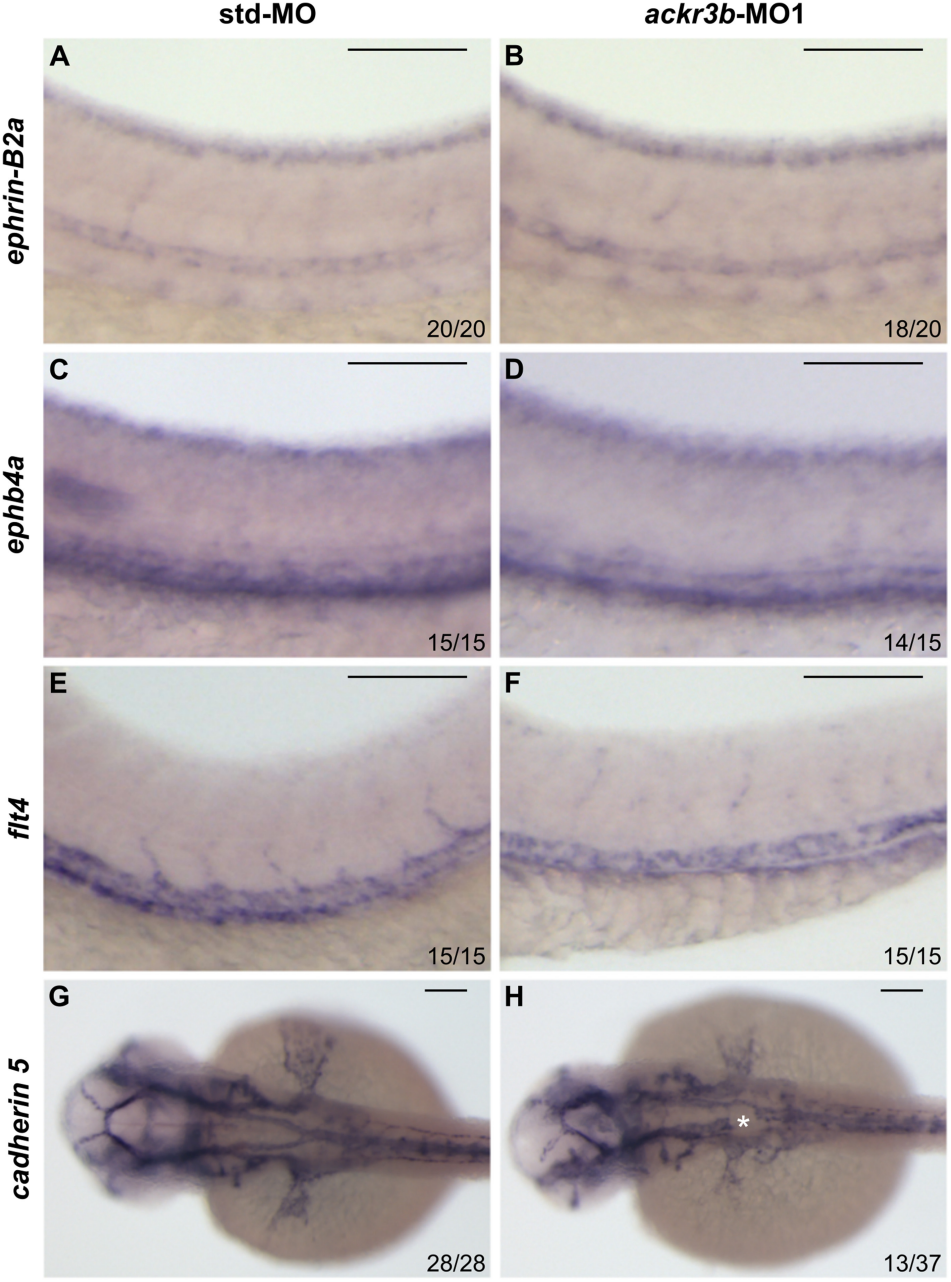
Effect of *ackr3b* knockdown on arterial/venous differentiation and lateral dorsal aorta (LDA) bifurcation. **(A–F)** WISH analysis of the arterial marker *ephrin-B2a* **(A,B)** and venous markers *ephb4a* **(C,D)** and *flt4* **(E,F)** expression was performed at 28 hpf on zebrafish embryos injected with std-MO **(A,C,E)** or *ackr3b-*MO1 **(B,D,F)**. Arterial and venous marker expression is normal in *ackr3b* morphants when compared to controls. **(G,H)** WISH analysis of *cadherin 5* expression was performed at 30 hpf on zebrafish embryos injected with std-MO **(G)** or *ackr3b*-MO1 **(H)** to investigate LDA formation. Note the interruption of LDA in *ackr3b* morphants [asterisk in **H**]. The number of embryos presenting the showed phenotype in respect to the total number of analyzed embryos is shown in each panel. Scale bar: 100 μm.

**Figure 4:**
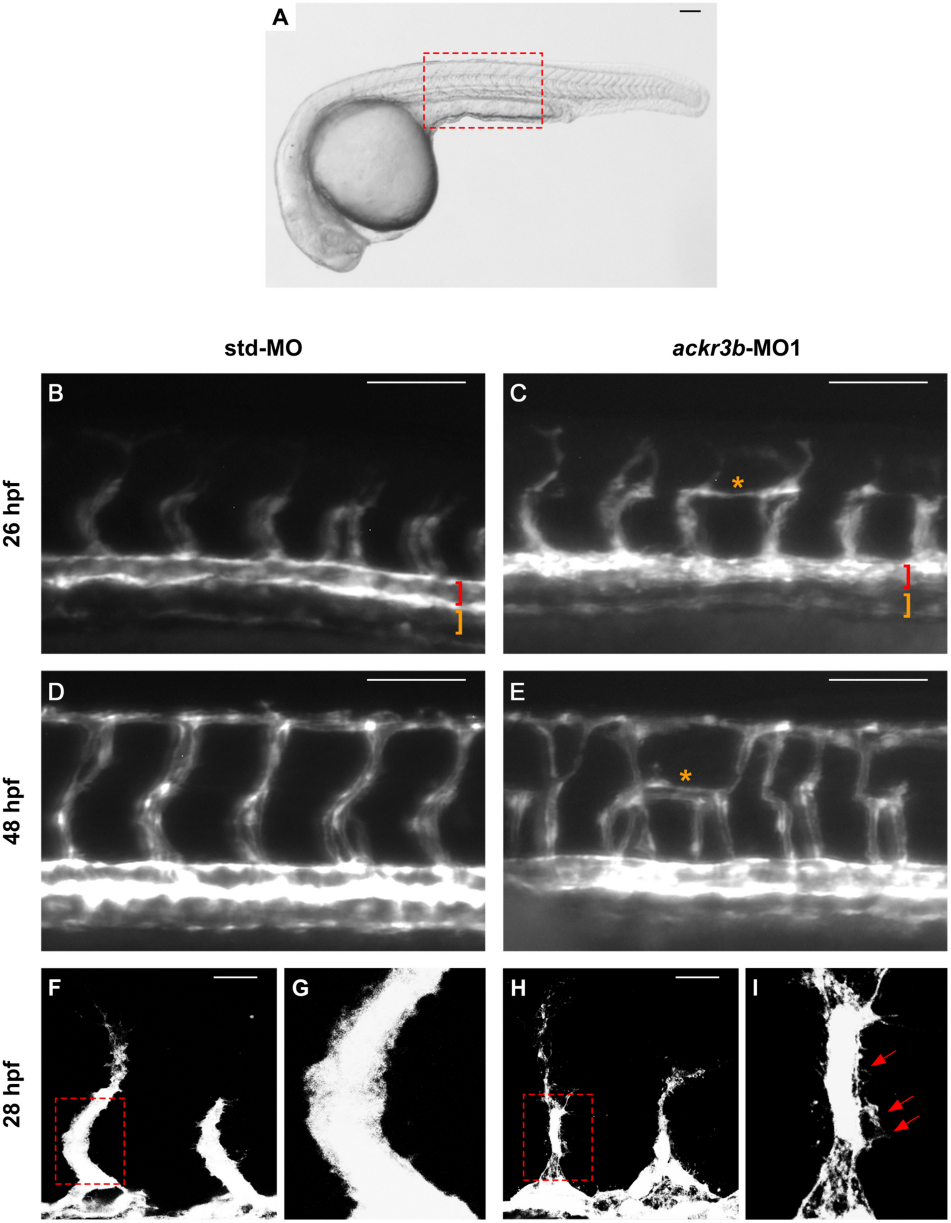
*ackr3b* knockdown affects ISV development and endothelial filopodia protrusions. *Tg(kdrl:EGFP)* embryos injected with std-MO (left panels) or *ackr3b*-MO1 (right panels) were photographed at 26 hpf **(B,C)** and 48 hpf **(D,E)** under an epifluorescence microscope. **(B–E)** High magnifications of the trunk region highlighted by the dotted box in **(A)** Asterisks in **(C**,**E)** indicate aberrant sprouts in *ackr3b*-MO1-injected embryos. Vasculogenesis occurs normally in *ackr3b* morphants as indicated by the presence of DA and PCV (brackets in **B,C**). **(F–I)** Confocal microscopy analysis of 28 hpf *Tg(kdrl:EGFP)* zebrafish embryos injected with std-MO **(F,G)** or *ackr3b-*MO1 **(H,I)**. Note the aberrant filopodia protrusions from stalk cells in *ackr3b* morphants (arrows in **I**) when compared to control embryos. **(G,I)** High magnification of the region highlighted by dotted boxes in **(F**,**H)**, respectively. **(A–E)** Scale bar: 100 μm. **(F–H)** Scale bar: 25 μm.

On this basis, *Tg(kdrl:EGFP)* transgenic zebrafish embryos, in which EGFP expression is driven by the promoter of the endothelial marker *kdrl*, were injected at the 1–4 cell stage with 0.2 pmoles/embryo of *ackr3b*-MO1 or control std-MO and the development of EGFP-labeled blood vessels was followed in live embryos. Vasculogenesis and early sprouting angiogenesis occur normally in *ackr3b* morphants as highlighted by the presence of the primary axial vessels and sprouting ISVs when compared to controls (brackets in Figures 4B,C). Accordingly, *ackr3b* downregulation does not affect the expression of the vascular endothelial growth factor *vegfa* at 16 hpf (data not shown) and of the specific arterial and venous markers *ephrin-B2a, ephb4a*, and *flt4* at 28 hpf (Figures 3A–F).

Despite the normal development of axial vessels and the presence of sprouting ISVs, *ackr3b* downregulation caused remarkable alterations of ISV patterning along the trunk and tail. Indeed, developing ISVs lose the correct direction of migration at 26 hpf in 76% of *ackr3b* morphants (58/76 *ackr3b*-MO1 vs. 6/70 std-MO; Fisher test: *p* < 0.0001 in four independent experiments) (Figures 4B,C). In addition, *ackr3b* morphants were characterized by the presence of ectopic and aberrant sprouts mostly localized at the level of the horizontal myoseptum (asterisk in Figure 4C). Moreover, confocal microscopy analysis revealed that ISVs of *ackr3b* morphants have persistent aberrant filopodia extensions in both stalk and tip cells when compared to controls (Figures 4F–I) (4/5 *ackr3b*-MO1 injected embryos vs. 1/7 *std*-MO injected embryos). Nevertheless, the dorsal longitudinal anastomotic vessels (DLAVs) that generate by the fusion of two adjacent ISVs appear to form normally at 48 hpf in both control and *ackr3b* morphants (Figures 4D,E). Of note, 33% of embryos injected with *ackr3b*-MO1 showed also alterations of LDA development (13/37 *ackr3b*-MO1 vs. 0/28 std-MO; Fisher test: *p* < 0.0001) (Figures 3G,H), in keeping with previous observations on *cxcr4a* morphants [see (9) and below].

**Figure 5:**
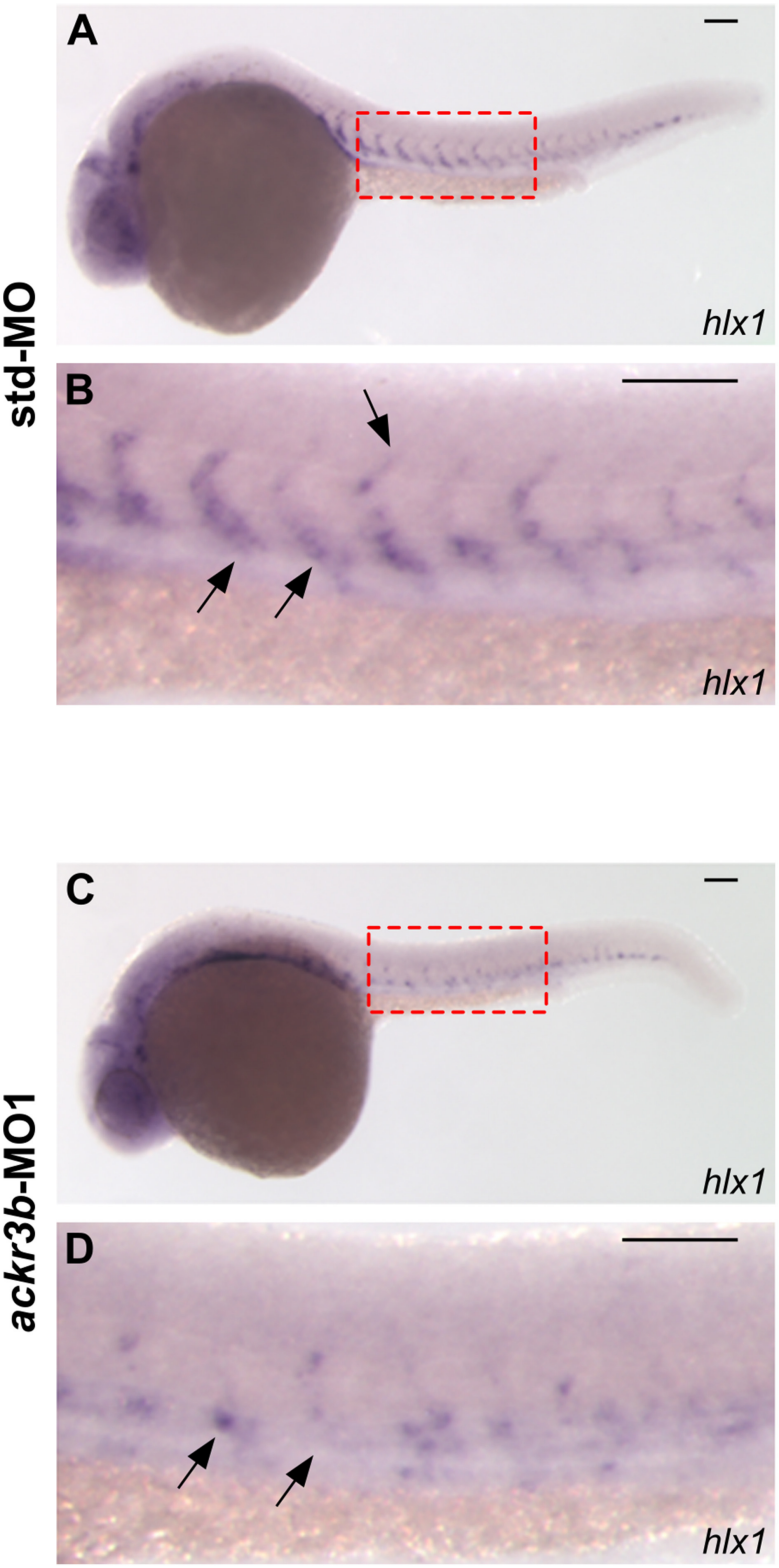
*ackr3b* knockdown impairs *hlx1* expression. **(A–D)** WISH analysis of *hlx1* expression in 26 hpf-embryos injected with std-MO **(A,B)** or *ackr3b-*MO1 **(C,D)**. **(B,D)** High magnification of the region highlighted by dotted boxes in **(A,C)**, respectively. *hlx1* is expressed by tip and stalk ISV endothelial cells in std-MO injected embryos (arrows in **B**) whereas its expression is downregulated in *ackr3b* knockdown embryos (arrows in **D**). Scale bar: 100 μm.

The homeobox gene *hlx1* is selectively expressed by tip and stalk endothelial cells during ISV sprouting in zebrafish embryo. In sprouting vessels, Hlx1 exerts a critical role in the maintenance of the stalk cell potential by repressing the migration of tip cells that express high levels of pro-migratory genes (31). Notably, *hlx1* expression in sprouting ISVs was significantly downregulated at 26 hpf in zebrafish embryos injected with *ackr3b*-MO1 when compared to controls (87/107 *ackr3b*-MO1 vs. 15/66 std-MO in four independent experiments; Fisher test: *p* < 0.0001) (Figures 5A–D).

In order to confirm the effects of *ackr3b* knockdown on ISV migration, zebrafish embryos were injected with *ackr3b*-MO2, a previously described MO directed against the 5′ UTR spanning the *ackr3b* ATG start codon (18) and distinct from *ackr3b*-MO1. When administered at an optimal dose equal to 1.2 pmoles/embryo, 56.5% of *ackr3b* morphants display lateral protrusions emerging from ISVs at 26 hpf (13/23 *ackr3b*-MO2 vs. 5/29 std-MO; Fisher test: *p* < 0.005). The effect persisted during embryo development, with 74% of morphants showing impaired ISV branching at 48 hpf (14/19 *ackr3b*-MO2 vs. 5/25 std-MO; Fisher test: *p* < 0.001). Thus, *ackr3b*-MO2 causes a phenotype similar to that observed with *ackr3b*-MO1, thus demonstrating the specificity of the effect.

**Figure 6:**
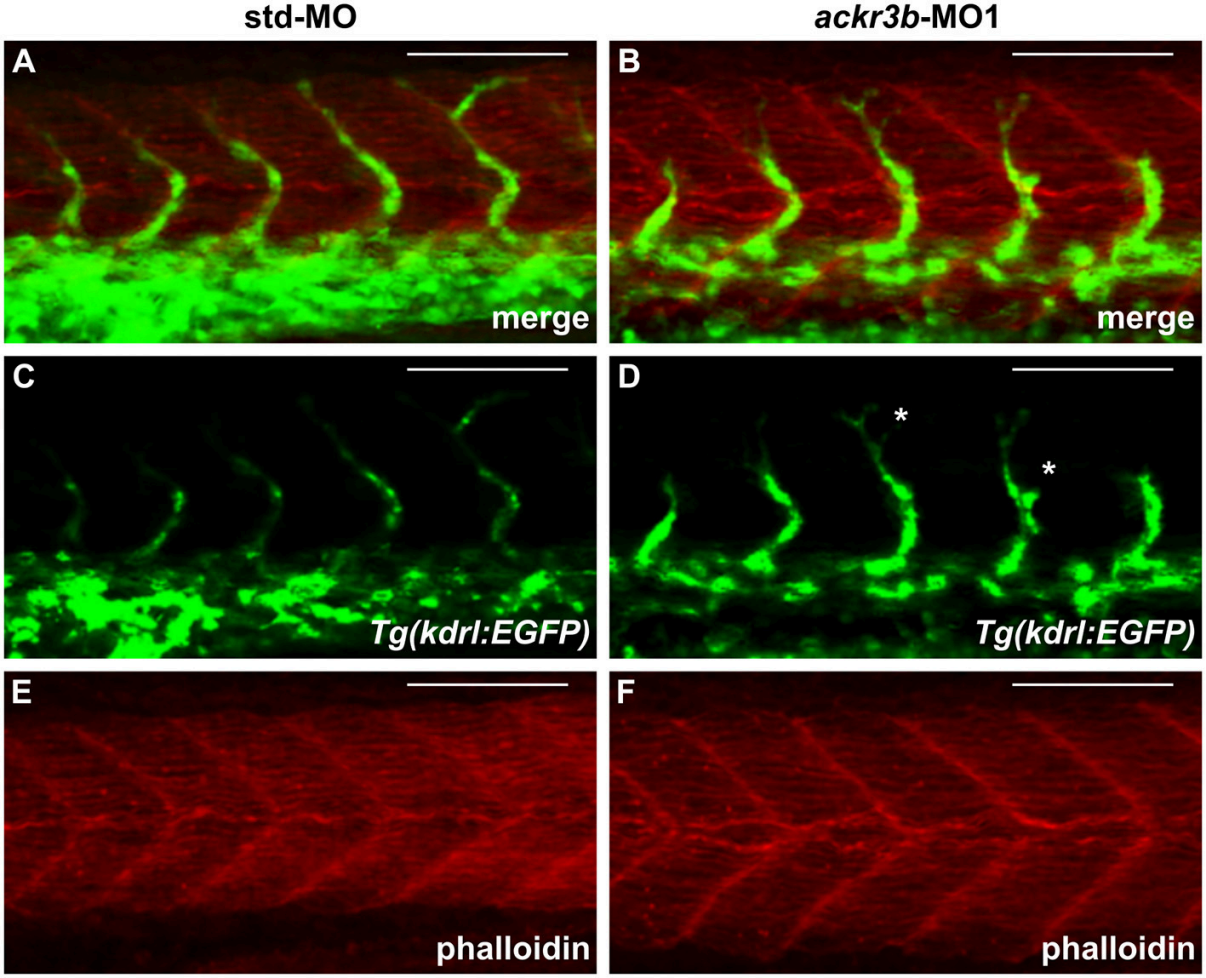
*ackr3b* knockdown does not affect somite boundary development. Fluorescence analysis of 28 hpf *Tg(kdrl:EGFP)* zebrafish embryos injected with std-MO **(A,C,E)** or *ackr3b-*MO1 **(B,D,F)** after phalloidin staining to reveal somitic actin fibers (red in **A,B,E,F**). Note that somitic boundary formation occurs normally in *ackr3b* morphants also in the presence of impaired ISV patterning (white asterisks in **D**). Scale bar: 100 μm.

As described above, *ackr3b* is expressed in somites during embryonic development. In order to rule out the possibility that the observed ISV mispositioning was due to somitic defects, phalloidin staining was performed on *ackr3b* morphants at 26–28 hpf. As shown in Figures 6A–F, no significant alterations of somitic boundary formation were observed in *ackr3b*-MO1-injected embryos with mild or close-to-normal phenotypes, including those embryos characterized by the presence of impaired ISVs (27/39 *ackr3b*-MO1 vs. 13/14 std-MO in two independent experiments).

**Figure 7:**
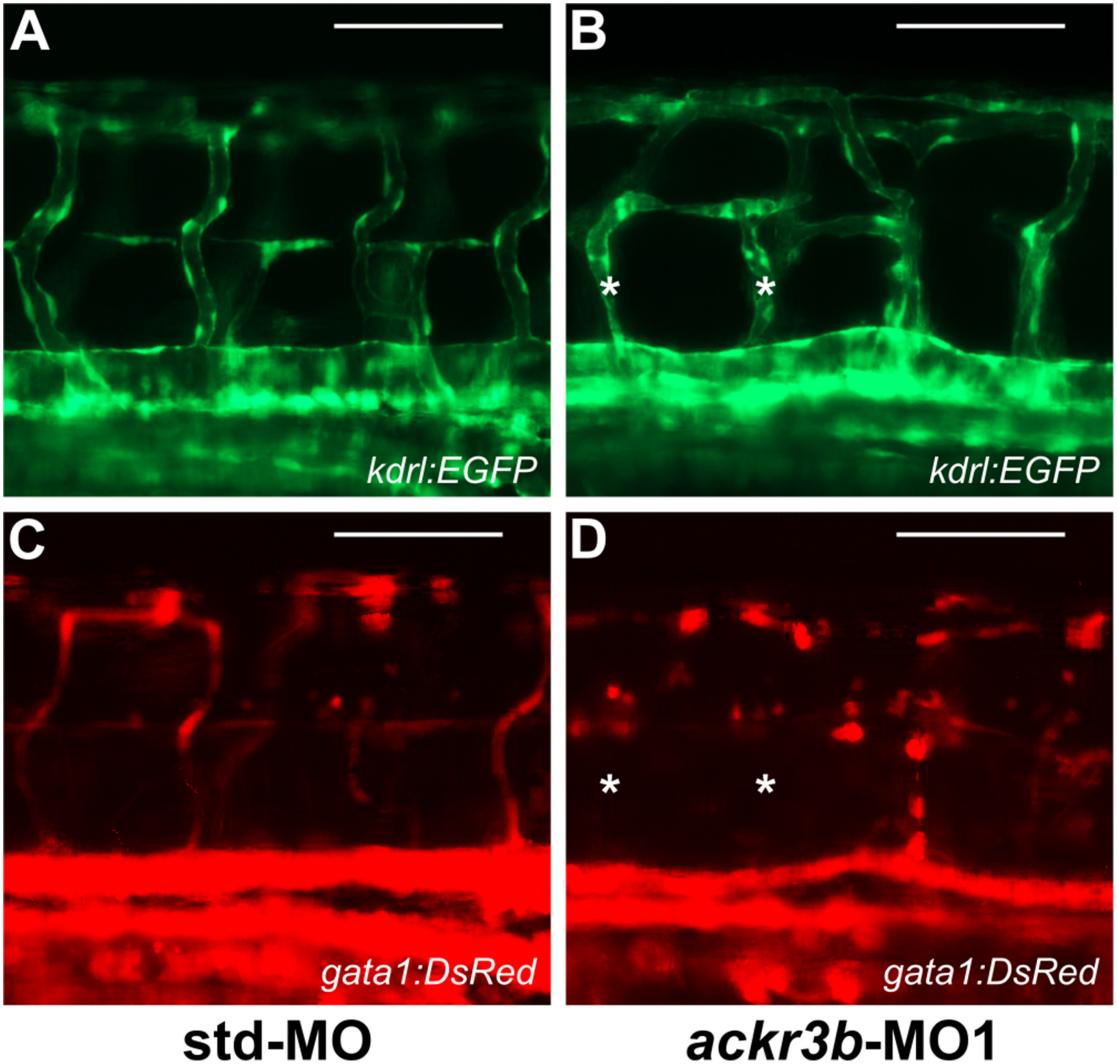
*ackr3b* knockdown causes alterations of blood circulation. **(A–D)** *Tg(kdrl:EGFP;gata1:DsRed)* embryos injected with std-MO **(A,C)** or *ackr3b*-MO1 **(B,D)** were photographed at 2 dpi under an epifluorescence microscope. Gata1^+^ elements were absent in Kdrl^+^ ISVs of *ackr3b* morphants (asterisks in **B,D**). Scale bar: 100 μm.

To investigate whether the vascular defects observed in *ackr3b* morphants were associated to significant alterations of blood circulation, double transgenic *Tg(kdrl:EGFP;gata1:DsRed)* zebrafish embryos that express EGFP in endothelial cells and DsRed in erythroid cells were injected with *ackr3b*-MO1 and assessed for the presence of gata1-positive circulating elements at 2 days post injection (2 dpi). Blood circulation was completely abrogated in 36% of *ackr3b* morphants and gata1-positive circulating cells were absent in the ISVs of 69% of the remaining *ackr3b* morphants that showed a normal axial circulation (11/16 *ackr3b*-MO1 vs. 3/43 std-MO; Fisher test: *p* < 0.0001) (Figures 7A–D). Similar results were obtained after *ackr3b*-MO2-injection (data not shown). These data support and extend previous observations about the defects in blood circulation in the trunk or tail of *ackr3b* morphants detectable after bloodstream injection of a FITC-dextran dye (13, 32).

**Figure 8:**
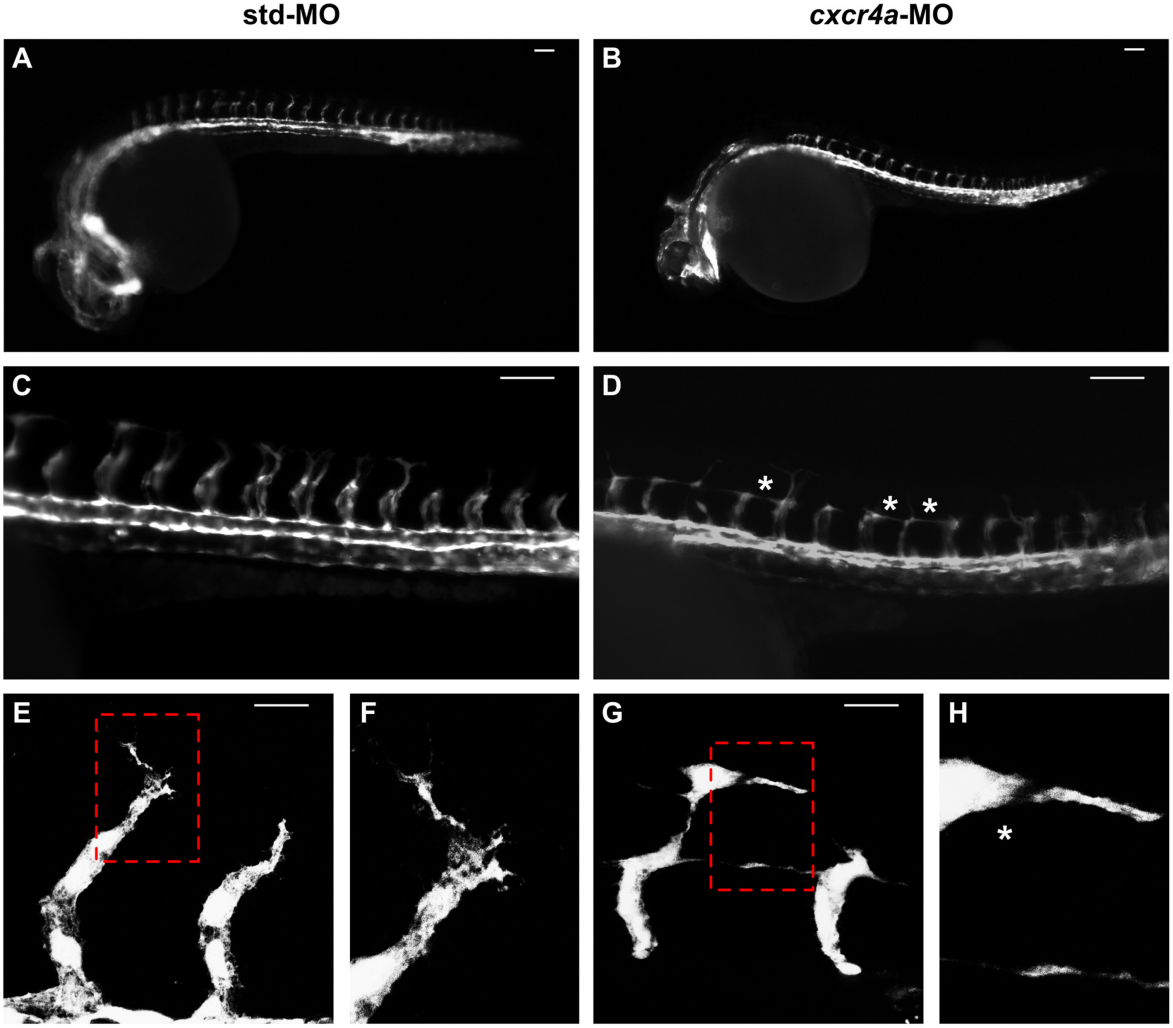
*cxcr4a* knockdown affects ISV patterning. *Tg(kdrl:EGFP)* embryos injected with std-MO **(A**,**C,E,F)** or *cxcr4a*-MO **(B,D,G,H)** were photographed at 26 hpf under an epifluorescence microscope. **(C,D)** Extended focus of z-stacks images representing high magnification of the trunk region. Asterisk in **(D)** indicates aberrant ISV branching in *cxcr4a*-MO-injected embryos. **(E–H)** Confocal microscopy analysis of 28 hpf *Tg(kdrl:EGFP)* zebrafish embryos injected with std-MO **(E,F)** or *cxcr4a-*MO **(G,H)**. Control embryos show filopodia protrusions emerging from sprouting ISVs **(E)**. At variance, filopodia protrusions are strongly reduced or absent in *cxcr4a* morphants **(**asterisk in **H)** despite ectopic ISV branching **(G)**. High magnification of the regions highlighted by dotted boxes are shown in **(E**,**G)**, respectively. **(A–D)** Scale bar: 100 μm. **(E–G)** Scale bar: 25 μm.

As described above, *cxcr4a* expression occurs in zebrafish endothelium of DA and developing ISVs between 20 and 26 hpf. Moreover, *cxcr4a* is expressed in LDA between 18 somites and 24 hpf (9). On these bases, in order to further assess a possible role of the Cxcr4/Ackr3 system in ISV guidance, *Tg(kdrl:EGFP)* zebrafish embryos were injected with 1.0 pmoles/embryo of a specific *cxcr4a*-MO. In agreement with previous observations (9), LDA interruption was present in 81% of *cxcr4a* morphants at 26 hpf (30/37 *cxcr4a*-MO in two independent experiments). Moreover, 70% of *cxcr4a* morphants with LDA interruption displayed alterations in ISV patterning resembling those observed in *ackr3b* morphants (21/30 *cxcr4a*-MO vs. 11/61 std-MO; Fisher test: *p* < 0.0001) (Figures 8A–D). However, at variance with *ackr3b* morphants, 43% of *cxcr4a*-MO-injected embryos (*n* = 7) showed only few or no filopodia protrusions in both stalk and tip ISV cells at 28 hpf when compared to controls (Figures 8E–H). These data are in keeping with previous observations showing that endothelial cell sprouting and filopodia extensions are severely impaired after blockage of CXCR4/CXCL12 axis in a murine model of retinal vascularization (8) and that filopodia are not essential for endothelial tip cell migration (33).

### ACKR3 Guides CXCL12-Mediated Endothelial Cell Migration

The above data point to a role for Ackr3b in shaping a Cxcl12 gradient for ISV guidance during zebrafish embryo development. CXCL12 is known to represent a CXCR4-mediated chemotactic stimulus for human endothelial cells (34). Previous studies had shown the possibility to evaluate the directional migration of bone marrow-derived dendritic cells in response to ACKR4-shaped CCL19 gradient using an *in vitro* co-culture assay (35). In this frame, to assess the capacity of ACKR3 to provide guidance cue for the migration of human endothelial cells, we developed an *in vitro* μ-slide cell co-culture chemotaxis assay (36) in which ACKR3 expression in CHO cells generate a CXCL12 gradient that guides HUVEC migration.

**Figure 9:**
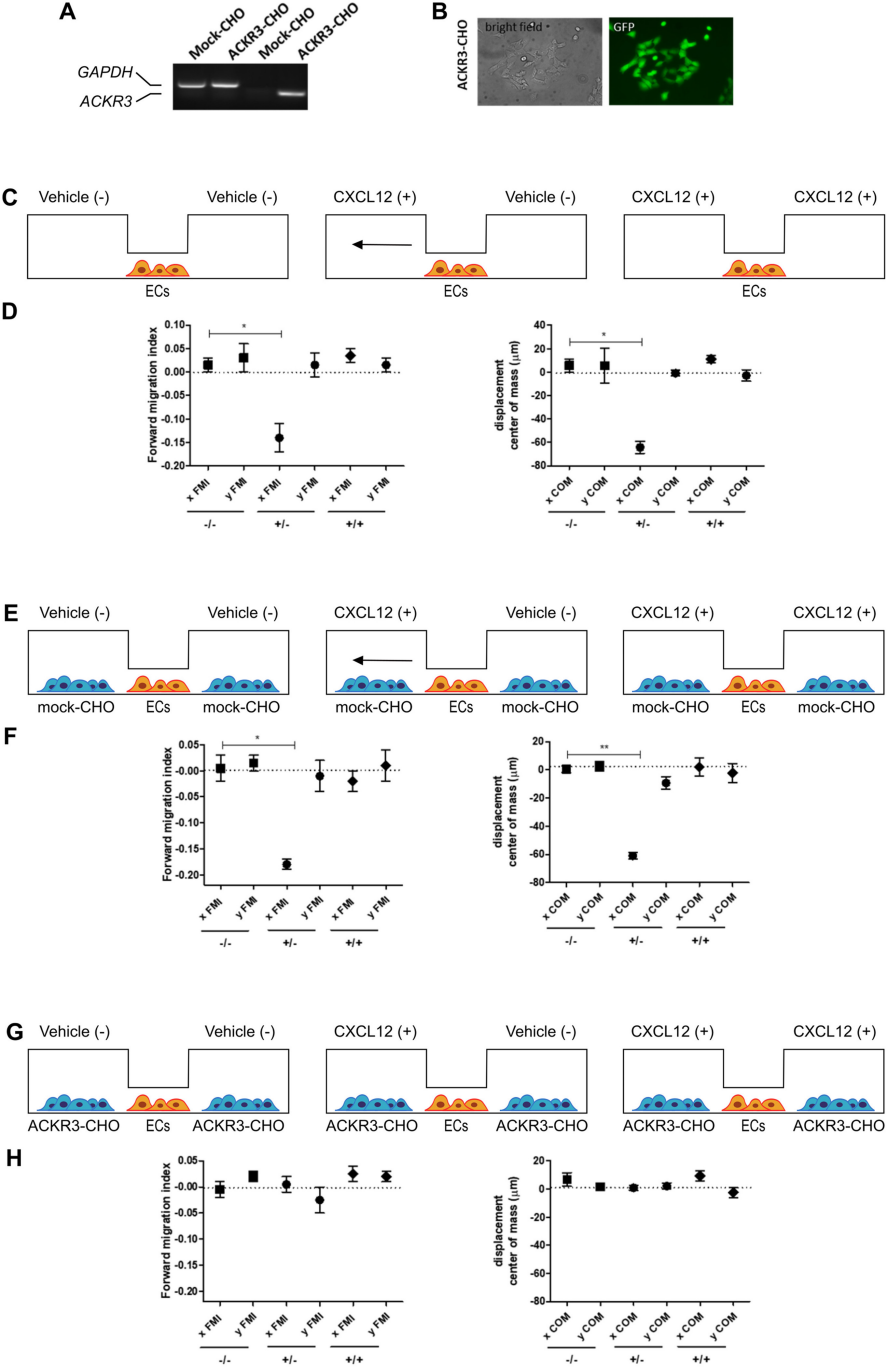
Setup of the μ-slide cell co-culture chemotaxis assay. **(A)** semi-quantitative PCR analysis of mock-CHO and ACKR3-CHO cells. **(B)** Microphotographs of EGFP-positive ACKR3-CHO cells. **(C)** Schematic representation of the μ-slide chamber. HUVECs (hereafter referred to as ECs) adherent to the central observation area are exposed to three different experimental conditions: no CXCL12 (–/–), addition of 50 ng/ml of CXCL12 to one (+/–) or both (+/+) lateral reservoirs. The horizontal arrow indicates the anticipated direction of HUVEC migration in the (+/–) experimental condition. **(D)** Quantification of forward migration index (FMI) and displacement of centre of mass (COM) parameters measured for HUVECs treated as illustrated in **(C)**. **(E)** Schematic representation of the μ-slide chamber in the presence of mock-CHO cells. HUVECs were seeded in the central observation area whereas mock-CHO cells were seeded in both lateral reservoirs. After 4 h, cells were left untreated (–/–) or were incubated with 50 ng/ml CXCL12 added to one (+/–) or both (+/+) lateral reservoirs. **(F)** At the end of the incubation, FMI and COM parameters were calculated. **(G)** Schematic representation of the μ-slide chamber in the presence of ACKR3-CHO cells. HUVECs were seeded in the central observation area whereas ACKR3-CHO cells were seeded in both lateral reservoirs. After 4 h, cells were left untreated (–/–) or were incubated with 50 ng/ml CXCL12 added to one (+/–) or both (+/+) lateral reservoirs. At the end of the incubation, FMI and COM parameters were calculated **(H)**. Data in panels **(D**,**F**,**H)** are the mean ± S.E.M of two independent experiments. ^*^p < 0.05 or better, Student *t*-test.

To this purpose, we transfected CHO cells with a bicistronic pIRES-EGFP vector harboring the human *ACKR3* cDNA to generate a stable EGFP-positive ACKR3-overexpressing CHO cell line (ACKR3-CHO cells, Figures 9A,B). Then, we performed a preliminary set of experiments to confirm the ability of CXCL12 to induce a chemotactic response in HUVECs in a μ-slide chemotaxis chamber that schematically consists of two lateral reservoirs and a central observation area that contains the cells under investigation (36). To this aim, HUVECs that physiologically express CXCR4 (37) were seeded in the central observation area of the chamber and exposed to an optimal concentration of CXCL12 that was loaded in one or both lateral reservoirs, as schematically illustrated in Figure 9C. As anticipated, CXCL12 induces a directional migratory response in HUVECs when loaded in one but not in both lateral reservoirs. As shown in Figure 9D, this was confirmed by a significant decrease of the two quantitative descriptive x-axis parameters “forward migration index” (xFMI, representing the efficiency of the forward migration of cells parallel to the gradient) and of the corresponding “displacement of center of mass” parameter (xCOM, representing the average of all cell positions at the end of the migration experiment) (36). This occurred in the absence of any change in the y-axis yFMI (representing the efficiency of cell migration perpendicular to the gradient) and the corresponding yCOM value (Figure 9D). A similar chemotactic response to CXCL12 gradient was observed when HUVECs were seeded in the central observation area and mock-CHO cells were seeded in both lateral reservoirs before CXCL12 addition in one of the two lateral chambers. In contrast, no directional migration was observed in the absence of CXCL12 or when both lateral reservoirs were filled with the chemokine solution in the presence of mock-CHO cells (Figures 9E,F).

**Figure 10:**
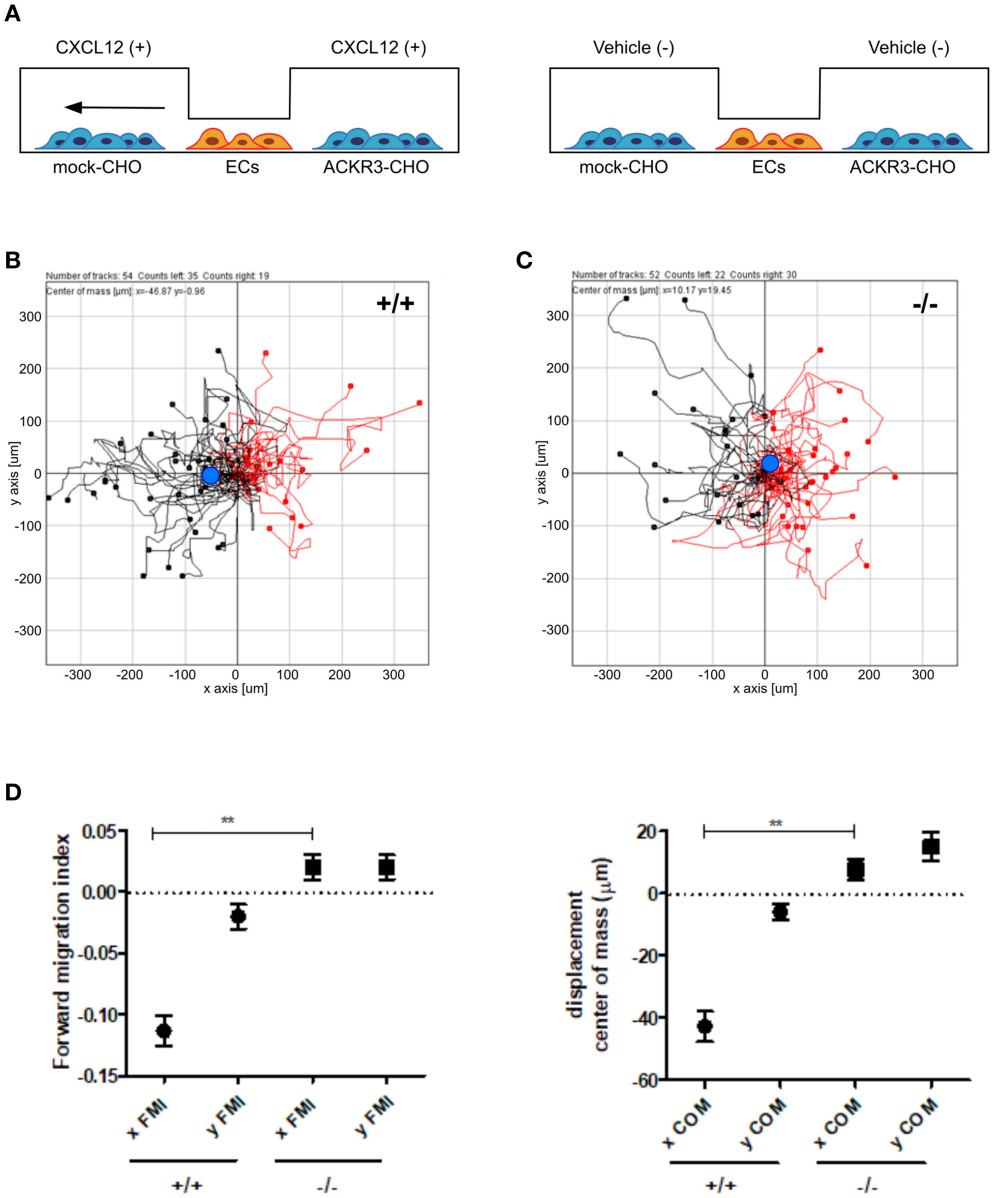
ACKR3 generates a chemotactic CXCL12 gradient for HUVECs. **(A)** Schematic representation of the μ-slide chamber cell co-culture chemotaxis assay. HUVECs (indicated as ECs) were seeded in the central observation area whereas mock- and ACKR3-CHO cells are seeded in the left and right lateral reservoirs, respectively. When CXCL12 is added to both reservoirs (left panel), ACKR3- CHO cells act as chemokine scavenger and generate a chemotactic CXCL12 gradient for HUVECs. No migration was observed when CXCL12 was replaced by vehicle (right panel). **(B,C)** Leftward (black symbols) and rightward (red symbols) trajectory plots of the migration of individual HUVECs exposed to the experimental conditions described in **(A)**. Blue dot: centre of mass. **(D)** Quantification of the corresponding forward migration index (FMI) and of the displacement of centre of mass (COM) parameters. Data represent the mean ± S.E.M. of three independent experiments. ^**^p < 0.01, Student *t*-test.

On this basis, to assess the impact of ACKR3-expressing CHO cells on CXCL12-mediated endothelial cell migration, HUVECs were seeded in the central observation area of the chamber whereas mock-CHO and ACKR3-CHO cells were seeded separately in the two lateral reservoirs in presence or in absence of the chemokine (Figure 10A). Under these experimental conditions, HUVECs migrate toward mock-CHO cells after the addition of CXCL12 in both reservoirs (Figures 10B–D). This indicates that the scavenging activity of ACKR3 expressed by CHO transfectants seeded on the opposite reservoir in respect to mock-CHO cells is able to create a directional CXCL12 gradient sensed by HUVECs at the center of the chamber. Accordingly, no directional migration of HUVECs was observed when ACKR3-CHO cells were seeded in both lateral reservoirs in the absence of CXCL12 or in the presence of the chemokine in one or both reservoirs, thus confirming the capacity of ACKR3 to affect the formation of an CXCL12 gradient (Figures 9G,H). See Table S1 for a schematic summary of the HUVEC migration responses to the various experimental conditions.

## Discussion

Previous observations had identified ACKR3 as a CXCL12 scavenging receptor responsible for the generation of guidance cues for CXCR4-expressing cells in different organ systems (2–4, 6, 9–11, 14, 16, 19, 22, 31, 38–40). Our data extend these observations by showing for the first time the capacity of this receptor to provide guidance cues also for endothelial cells during developmental angiogenesis in zebrafish. Indeed, in the present work, we provide evidence for a role of *ackr3b*, a zebrafish co-ortholog of human *ACKR3*, in establishing endothelial cell migration cues during the angiogenic process in ISVs and LDA of zebrafish embryos. Accordingly, we demonstrate the ability of ACKR3 to create a directional CXCL12 gradient sensed by CXCR4-expressing HUVECs in an *in vitro* cell co-culture chemotaxis assay.

The angiogenic process in the trunk of zebrafish embryos starts at about 22 hpf (39), in a time window partially overlapping the expression of *cxcr4a, cxcl12a/b*, and *ackr3b*. During zebrafish somitogenesis, *cxcr4a* and its ligands *cxcl12a* and *cxcl12b* are expressed in the somites (29). At following developmental stages, *cxcl12a* is expressed in the horizontal myoseptum whereas *cxcl12b* in DA (38). Starting from 20 ss, *cxcr4a* expression is restricted to the most caudal somites, being detectable in the DA and endothelial cells that form the ISVs from 24 (11) to 26 hpf. Similarly, in agreement with previous observations (28), WISH and analysis of coronal and transverse cross sections of 18 ss, 20 hpf, and 26 hpf embryos revealed a tightly regulated time-dependent pattern of *ackr3b* expression in zebrafish somites. Notably, *vegfa*, that plays a pivotal role in vasculogenesis and angiogenesis in zebrafish, is expressed in the somites at the same developmental stages, thereby inducing the expression of genes that will be involved in arteriovenous differentiation (41) and ISV sprouting few hours later (40). Moreover, recent data have demonstrated that *vegfa* overexpression or deficiency induce loss of endothelial cell filopodia extension, thereby altering ISV pathfinding, in a tight time-dependent manner (20), thus indicating a fine-tuned regulation of the angiogenic process of the developing vessels of the trunk of zebrafish embryos. Together, these observations reinforce the hypothesis that Ackr3b may play a role in the interplay between Cxcr4a and Cxcl12 during vascular development in this district.

Our data show that ISV development is severely impaired in *ackr3b* morphants: ISVs lose the correct direction of migration at 26 hpf, form aberrant sprouts at the level of the horizontal myoseptum and show persistent filopodia protrusions in both ISV stalk and tip cells. Accordingly, *ackr3b* downregulation causes a significant alteration of the expression of the ISV tip and stalk cell marker *hlx1* (31). In parallel with the defects observed in ISV development, *ackr3b* knockdown impairs the bifurcation process of LDA, a phenotype that characterizes also *cxcr4a* morphants and mutants (9). Notably, these alterations occurred in the absence of major defects of the vasculogenic process, as demonstrated by the normal development of DA and PCV following *ackr3b* downregulation, and were paralleled by the lack of *gata1*-positive circulating blood cells even in the ISVs of *ackr3b* morphants with a normal axial circulation.

**Figure 11:**
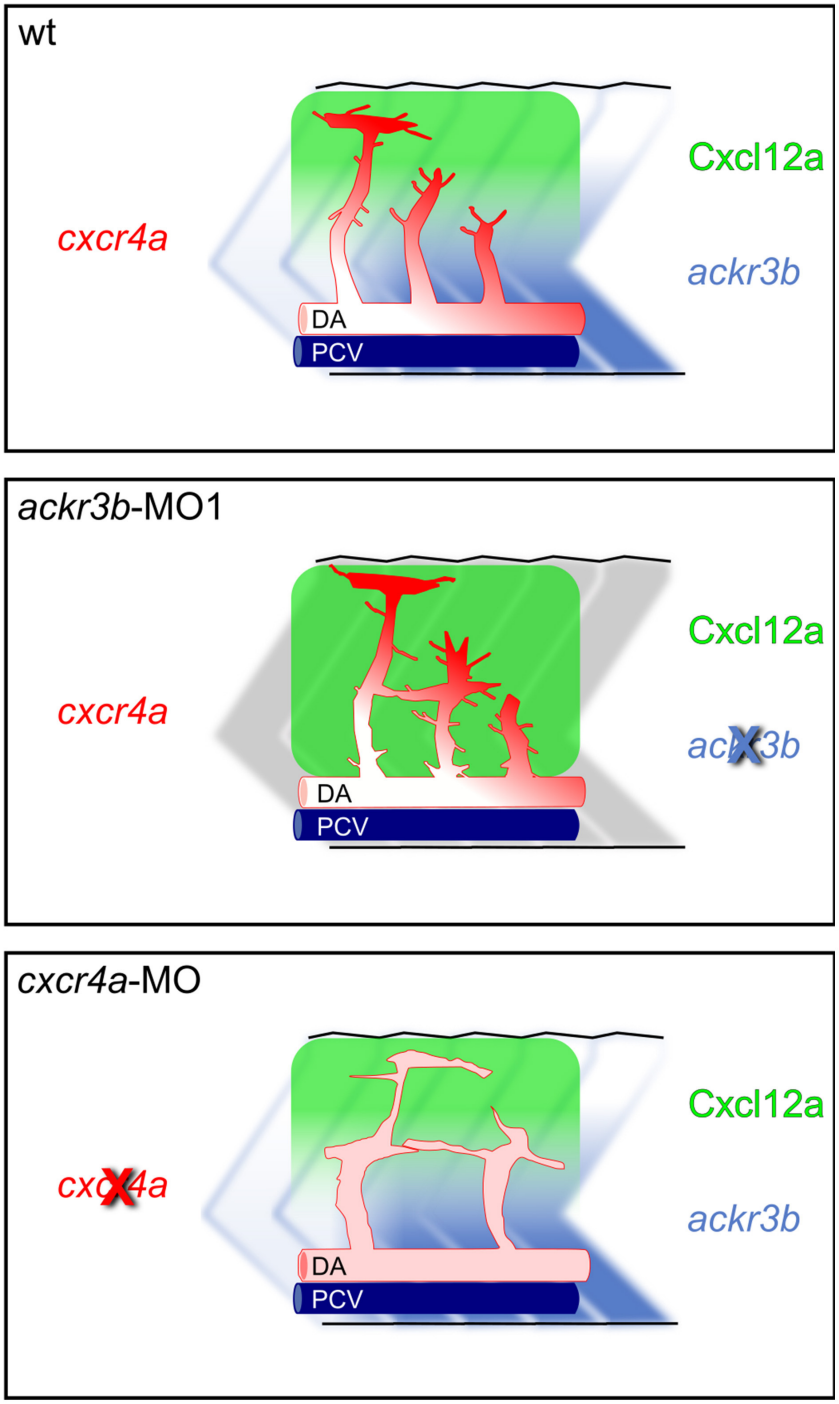
Role of Ackr3b during the angiogenic ISV sprouting process in zebrafish embryos. In a physiological situation (**top panel**), *ackr3b* expression generates the Cxcl12a gradient that guides the sprouting of ISV endothelial cells. Tip cell guidance is mediated by the receptor Cxcr4a, which is mainly expressed on filopodia. Knockdown of *ackr3b* (**middle panel**) disrupts the Cxcl12a gradient and sprouting endothelial cells maintain a tip cell-like phenotype, characterized by abnormal filopodia protruding from the whole vessel. The sprouting process results in the formation of aberrant sprouts localized in the region of the horizontal myoseptum. Knockdown of *cxcr4a* (**bottom panel**) hampers the response of sprouting endothelial cells to the Cxcl12a gradient, mimicking the phenotype observed in *ackr3b* morphants except for the strong reduction in filopodia protrusion both in tip and stalk cells.

The hypothesis that Ackr3b may play a role in the interplay between Cxcr4a and Cxcl12 during ISV development is further supported by the vascular defects we observed in *cxcr4a* morphants. Indeed, *cxcr4a* MO-injected embryos show altered ISVs that have lost the correct direction of migration, with a phenotype resembling that observed after *ackr3b* knockdown. Notably, at variance with the ISVs of *ackr3b* morphants, downregulation of *cxcr4a* expression results in a strong reduction in filopodia extensions in ISV stalk and tip cells. These data are in agreement with previous observations in a murine model of retinal angiogenesis (8) and support the hypothesis that filopodia might be dispensable for endothelial cell migration (33). Nevertheless, ISV endothelial cells fail to sense Cxcl12 guidance cues in the absence of Cxcr4a, thus acquiring a disorganized sprouting behavior. Accordingly, *ackr3b* knockdown affects the formation of a guiding Cxcl12 gradient, leading to an undirected ISV migratory response characterized by abnormal filopodia protrusions originating from both tip and stalk cells. These findings are in keeping with the observation that filopodia distribution and dynamics depend on chemokine gradients in zebrafish embryo (42). See Figure 11 for a graphical representation of the effect of *ackr3b* and *cxcr4a* downregulation on Cxcl12 gradient formation and ISV development in the trunk of zebrafish embryo.

Previous observations have shown that ACKR3 blockade may increase the serum levels of CXCL12 in mice, possibly due to a reduced scavenging of the chemokine (43, 44). In addition, ACKR3 downregulation/inactivation, with consequent excess of CXCL12, may cause the loss of CXCR4 due to an increased ligand-dependent internalization and intracellular degradation of the receptor (45, 46). Thus, the possibility exists that an upregulation of systemic Cxcl12 levels may occur also in *ackr3b* zebrafish morphants and may contribute, together with the loss of guidance cues, to the aberrant filopodia extensions observed in tip and stalk cells because of an altered *cxcr4* expression and activation. Further experiments will be required to clarify this point.

In keeping with a role for *ackr3b* in endothelial cell guidance in zebrafish embryos, CXCL12 gradient generated by ACKR3 expression in CHO cells drives human endothelial cell migration in an *in vitro* cell co-culture chemotaxis assay. In this model, CXCR4-expressing HUVECs are seeded in the central observation area of a μ-slide chemotaxis chamber (36). As anticipated, HUVECs migrate directionally in response to CXCL12 added to one of the two lateral chambers, whereas no directional migration was observed when the chemokine was added to both chambers. At variance, the CXCL12 scavenging activity exerted by ACKR3-overexpressing CHO cells seeded in one of the two lateral reservoirs generates endothelial guidance cues in response to CXCL12 also when the chemokine was added simultaneously to both lateral chambers. No directional migration was instead observed in the absence of the chemokine or when mock or CHO transfectants were seeded in both lateral chambers.

*ACKR3* upregulation may occur in activated endothelial cells and may contribute to tumor angiogenesis (47). In addition, recent observations have shown that endothelial ACKR3 may drive CXCL12-mediated angiogenic responses *via* activation of the Akt signaling pathway (48). However, our data indicate that CXCL12 stimulation is not *per se* sufficient to trigger a directional migratory response in endothelial cells unless exposed to a concentration gradient of the chemokine as that generated by the expression of this chemokine scavenging receptor in adjacent cells.

In conclusion, our data demonstrate that Ackr3b mediates the guidance of sprouting endothelial cells during the angiogenetic development of ISVs and LDA in zebrafish embryos. Accordingly, our cell co-culture chemotaxis model indicates that the scavenging activity of ACKR3 is able to create a directional CXCL12 gradient sensed by human endothelial cells. Together, these findings offer novel information about the complexity of the signaling network that orchestrates sprouting angiogenesis, indicating that the CXCR4/CXCL12/ACKR3 interplay provides non-redundant guidance cues for developing blood vessels.

## Ethics Statement

All applicable international, national, and/or institutional guidelines for the care and use of animals were followed. Current national legislation do not require approval for research on zebrafish embryos.

## Author Contributions

Experiment conception and design: CT and MP. Experiment performing: CT, PC, AB, SB, EF, and JG. Data analysis: CT and MP. Analysis tools contribution: SM and GB. Manuscript preparation: CT and MP.

### Conflict of Interest Statement

The authors declare that the research was conducted in the absence of any commercial or financial relationships that could be construed as a potential conflict of interest.
