# Supplementary material for: Atypical Chemokine Receptor 3 Generates Guidance Cues for CXCL12-Mediated Endothelial Cell Migration
Source: Front Immunol. 2019 May 15;10:1092. doi: 10.3389/fimmu.2019.01092 (PMC6529557; doi:10.3389/fimmu.2019.01092)
Supplement: Supplementary file 1 [file Data_Sheet_1.PDF]

Supplementary Material

A

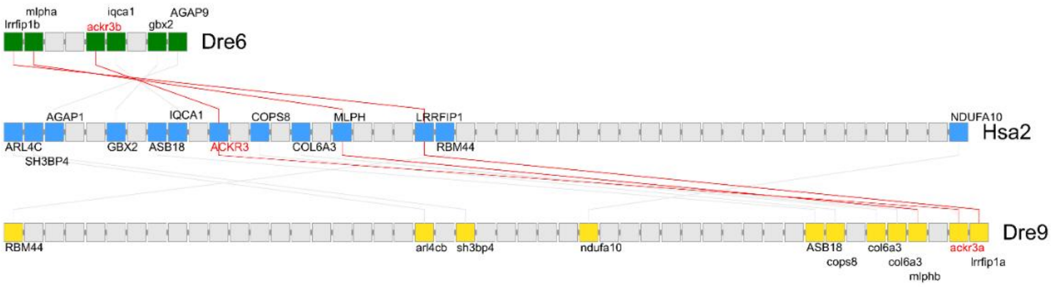

B

```
ACKR3           MDLHLFDYSEP-GNFSDISWPCNSSDCIVVDTVMCPNMPNKSVLLYTLSFIYIFIFVIGM    59
Ackr3a          -------------MWEELNFTDSNNNSSRVEAQMCPASFNRSALLNAMCTLYAFIFVVGL    47
Ackr3b          MSVNVNDFNDILDALGELNFSTLDDNVS--HVEVCHSTFSQRALLYALSVLYIFLFIIGL    58
                                ::.:   ..:    .. :*    .: .** ::. :* *:*::*:

ACKR3           IANSVVVWVNIQAKTTGYDTHCYILNLAIADLWVVLTIPVWVVSLVQHNQWPMGELTCKV    119
Ackr3a          AANALVVWVNMRSQRHYHETHMYILNLAVADLCVVATLPVWVSSLAQGGHWAFGQAACKL    107
Ackr3b          AANALVVWVNVRAERTRYETHLYILNLAIADLCVVATLPVSISSLLQLGHWPFGGAMCKI    118
                 **::*****::::   ::** ******:*** ** *:** : ** * .:* :*   **:

ACKR3           THLIFSINLFGSIFFLTCMSVDRYLSITYFTNTPSSRKKMVRRVVCILVWLLAFCVSLPD    179
Ackr3a          THLLFSVNLFASIFFLTCMSVDRYLSVVRFREISHRRGRQVRRLVCAITWLLALFASVPD    167
Ackr3b          THLIFSVNLFSSIFFLTCMSVDRYLSVKLFGDTPSQRKRRTRQIICVGVWLLALIAALPE    178
                ***:**:***.***************:  * :    * : .*:::*  .****: .::*:

ACKR3           TYYLKTVTSASNNETYCRSFYPEHSIKEWLIGMELVSVVLGFAVPFSIIAVFYFLLARAI    239
Ackr3a          TYFLRSVKSQYSHVTLCLPVYPEDNPLQWMVGIQLSFVVLGFAIPFPIIAVSYALLANTL    227
Ackr3b          IYFLQAEKSDHSDAIVCKAVYPVESMKEWTVGIQMSFFMLGFAIPFPVIAVFYVLLANTI    238
                 *:*:: .*  ..   *  .** ..  :* :*:::  .:****:** :*** * ***.::

ACKR3           SA-------SSDQEKHSSRKIIFSYVVVFLVCWLPYHVAVLLDIFSILHYIPFTCRLEHA    292
Ackr3a          ASTSTHSPSNGDQDRSVSKKVILMYIVVFIACWAPYHAVLLADALAMLGVLPLGCSSENG    287
Ackr3b          HP-------SVDQERRISRHLIFTYIVVFLVCWLPYHGALLLDTLAFLNVLPFNCTLENA    291
                         . **::  *:::*: *:***:.** *** .:* * :::*  :*: *  *:.

ACKR3           LFTALHVTQCLSLVHCCVNPVLYSFINRNYRYELMKAFIFKYSAKTGLTKLIDASRVSET    352
Ackr3a          LFVALHLTQCLSLLHCCVNPVVYSFAHRHYRYDLMKAFIFKYSTRTGLARLIDGSQGIET    347
Ackr3b          LYAALHLTQCFSLFHCCANPIIYNFINKNYRYDLMKAFIFKYSTKTGLARLIDASHVSET    351
                *:.***:***:**.***.**::*.* :::***:**********::***::***.*:  **

ACKR3           EYSALEQSTK---      362
Ackr3a          EYAMVENNPPTVT      360
Ackr3b          EYSAVENQGPL--      362
                **: :*:.
```

C

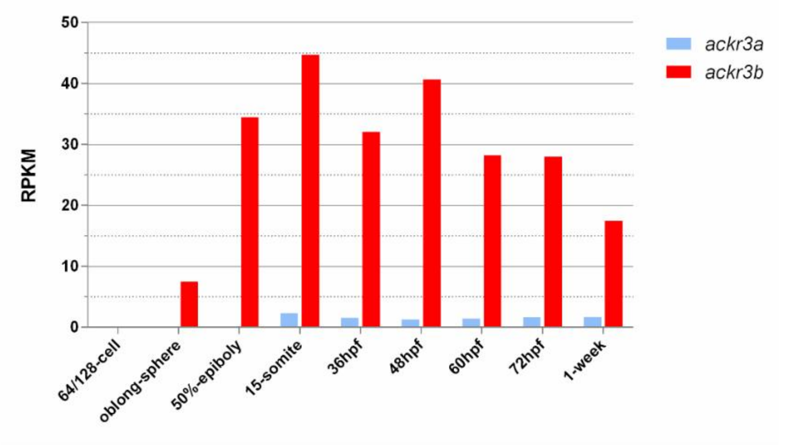

Fig. S1. Analysis of *ackr3* genes in zebrafish. A Composite cluster representation of conserved synteny around the *ACKR3* locus between *Danio rerio* chromosomes 6 (Dre6) and 9 (Dre9) and *Homo sapiens* chromosome 2 (Hsa2) generated using the Synteny Database (50-gene sliding window). Genes are drawn as squares with their order but not their physical location preserved. Colored squares are members of the cluster while grey squares represent genes in the interval that do not have orthologs (or paralogs) in the other segments. Red lines connect orthologous genes within the 3 clusters, gray lines identify orthologous genes between Hsa2 and either Dre6 or Dre9. Six gene pairs can be identified in orthologous pairwise clusters between Hsa2 and Dre6, and 11 gene pairs are present when considering Hsa2 and Dre9. The analysis has been performed based on *Danio rerio* Zv9 and *Homo sapiens* GRCH 37 genome assemblies. B Multiple sequence alignment of human ACKR3, zebrafish Ackr3a and zebrafish Ackr3b polypeptides obtained using the MUSCLE software. In the ClustalW output format, asterisks indicate positions which have a single, fully conserved residue, colons indicate conservation between groups of strongly similar properties while periods indicates conservation between groups of weakly similar properties. Residues are colored according to their physicochemical properties. **C** RNA-Seq data of *ackr3a* (blue bars) and *ackr3b* (red bars) expression at different stages of zebrafish development derived from a systematic study aimed at capturing the transcriptome landscape of zebrafish embryos and larvae [PMID: 23700457]. Gene expression levels are expressed as RPKM (Reads Per Kilobase per Million mapped reads).

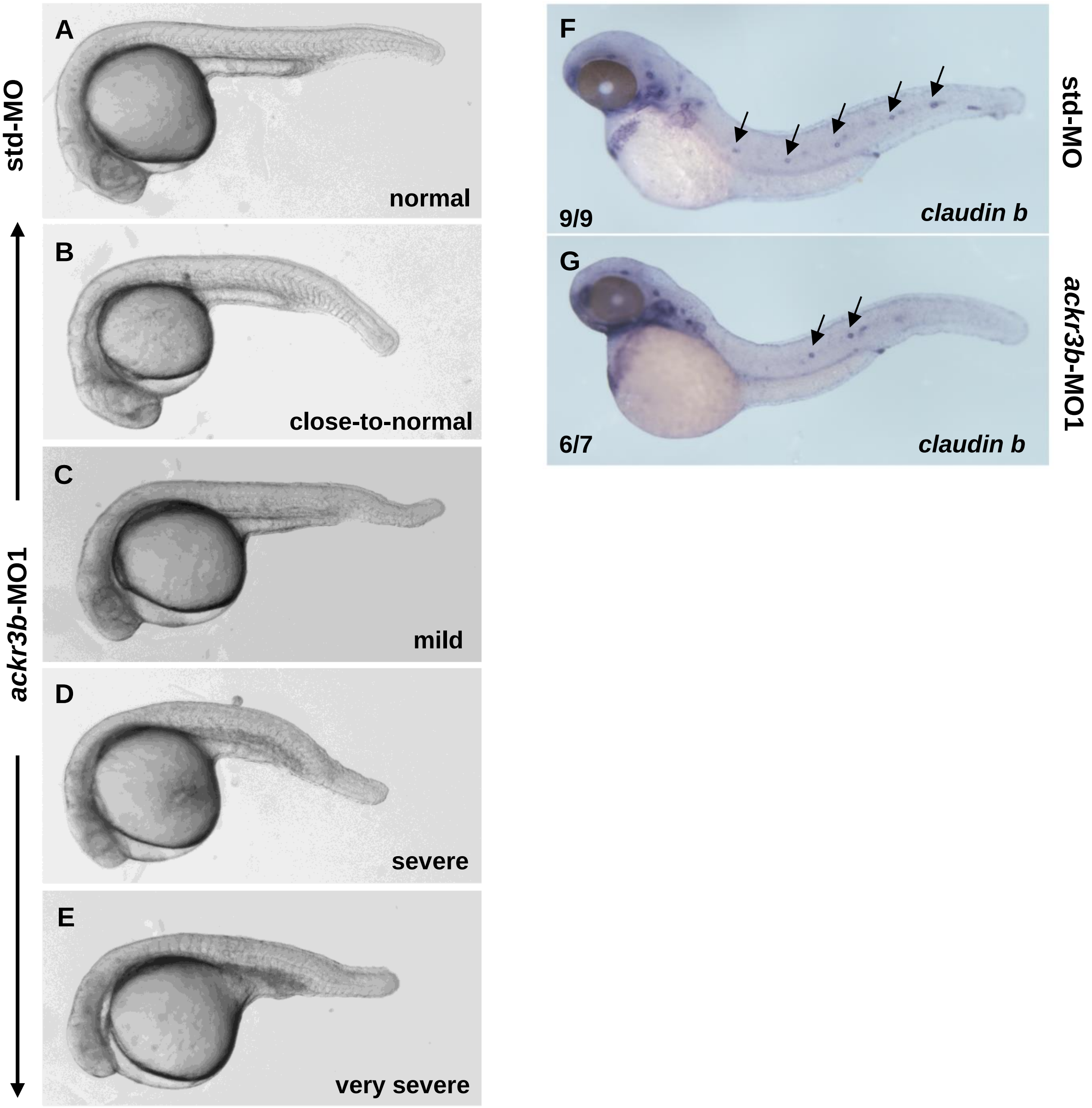

Fig. S2. Effect of *ackr3b* knockdown on zebrafish embryo development. A-E Embryos injected with 0.2 pmoles/embryo of std-MO (**A**) or *ackr3b*-MO1 (**B-E**) were photographed at 28 hpf. Embryos with close-to-normal (**B**) and mild (**C**) phenotype were used for all further analyses whereas severe (**D**) and very severe (**E**) phenotypes were discarded. **F, G** Effect of *ackr3b* knockdown on neuromast migration. WISH analysis of *claudin b* expression was performed at 48 hpf on zebrafish embryos injected with std-MO (**F**) or *ackr3b*-MO1 (**G**) to assess actual *ackr3b* knockdown. Note the impaired neuromast migration in *ackr3b* morphants (arrows in **G**) when compared to the normal positioning observed in control embryos (arrows in **F**). The number of embryos presenting the showed phenotype in respect to the total number of analyzed embryos is shown in both panels.

**Cells**	**- | ECs | -**			**mock-CHO | ECs | mock-CHO**			**ACKR3-CHO | ECs | ACKR3-CHO**			**mock-CHO | ECs | ACKR3-CHO**	
**CXCL12**	-/-	+/-	+/+	-/-	+/-	+/+	-/-	+/-	+/+	-/-	+/+
**Migration**	**No**	**Yes** ←	**No**	**No**	**Yes** ←	**No**	**No**	**No**	**No**	**No**	**Yes** ←

Table S1. Summary of the results obtained with the µ-slide cell co-culture chemotaxis assay. HUVECs (indicated as ECs) were seeded in the central observation area in the absence or presence of mock-CHO or ACKR3-CHO cells in the lateral reservoirs as indicated. Next, cells were exposed to three different experimental conditions: no CXCL12 (-/-) or addition of 50 ng/ml of CXCL12 to one (+/-) or both (+/+) lateral reservoirs. When present, arrow indicates the direction of HUVEC migration. No: no directional migration.
